# Mobile phones in cryptogenic strOke patients Bringing sIngle Lead ECGs for Atrial Fibrillation detection (MOBILE-AF): study protocol for a randomised controlled trial

**DOI:** 10.1186/s13063-017-2131-0

**Published:** 2017-08-29

**Authors:** Roderick W. Treskes, Willem Gielen, Marieke J. Wermer, Robert W. Grauss, Anouk P. van Alem, Reza Alizadeh Dehnavi, Charles J. Kirchhof, Enno T. van der Velde, Arie C. Maan, Ron Wolterbeek, Onno M. Overbeek, Martin J. Schalij, Serge A. Trines

**Affiliations:** 1 Department of Cardiology, Leiden University Medical Center, Albinusdreef 2, 2333 ZA Leiden, The Netherlands; 2 Department of Cardiology, Regionshospitalet Herning, Herning, Denmark; 3 Department of Neurology, Leiden University Medical Center, Leiden, The Netherlands; 4 Department of Cardiology, Haaglanden Medical Center, The Hague, The Netherlands; 5 Deparment of Cardiology, Groene Hart Hospital, Gouda, The Netherlands; 6 Department of Cardiology, Alrijne Hospital, Leiderdorp, The Netherlands; 7 Department of Medical Statistics, Leiden University Medical Center, Leiden, The Netherlands

## Abstract

**Background:**

Recently published randomised clinical trials indicate that prolonged electrocardiom (ECG) monitoring might enhance the detection of paroxysmal atrial fibrillation (AF) in cryptogenic stroke or transient ischaemic attack (TIA) patients. A device that might be suitable for prolonged ECG monitoring is a smartphone-compatible ECG device (Kardia Mobile, Alivecor, San Francisco, CA, USA) that allows the patient to record a single-lead ECG without the presence of trained health care staff. The MOBILE-AF trial will investigate the effectiveness of the ECG device for AF detection in patients with cryptogenic stroke or TIA. In this paper, the rationale and design of the MOBILE-AF trial is presented.

**Methods:**

For this international, multicentre trial, 200 patients with cryptogenic stroke or TIA will be randomised. One hundred patients will receive the ECG device and will be asked to record their ECG twice daily during a period of 1 year. One hundred patients will receive a 7-day Holter monitor.

**Discussion:**

The primary outcome of this study is the percentage of patients in which AF is detected in the first year after the index ischaemic stroke or TIA. Secondary outcomes include markers for AF prediction, orally administered anticoagulation therapy changes, as well as the incidence of recurrent stroke and major bleeds. First results can be expected in mid-2019.

**Trial registration:**

ClinicalTrials.gov, ID: NCT02507986. Registered on 15 July 2015.

**Electronic supplementary material:**

The online version of this article (doi:10.1186/s13063-017-2131-0) contains supplementary material, which is available to authorized users.

## Background

In approximately 30% of all patients with ischaemic stroke or transient ischaemic attack (TIA), no cause can be determined after standard evaluation. These strokes and TIAs are referred to as cryptogenic [1]. One of the known risk factors for ischaemic stroke is atrial fibrillation (AF) [2]. Current guidelines state that orally administered anticoagulation therapy (OAC) should be prescribed in case AF of any duration above 30 s is detected after ischaemic stroke/TIA on a 12-lead electrocardiogram (ECG) or lasting at least 30 s [3] on a 24-h Holter monitor [3, 4]. However, as AF is often asymptomatic and paroxysmal, [5] the detection of AF with the currently advised standard work-up using a 24-h Holter recording is low [6, 7].

Consequently, recently published research indicates that AF episodes may be missed using this standard evaluation. In the CRYSTAL-AF [6] and EMBRACE trials, [7] it was demonstrated that prolonged ECG monitoring yielded significantly higher percentages of detected AF in patients with cryptogenic stroke or TIA [6, 7].

It has been discussed that it is uncertain if subclinical AF, especially short episodes, require anticoagulation in patients with cryptogenic stroke [8]. Several trials (NCT02313909, NCT02427126 and NCT02239120) have started to randomise patients with cryptogenic stroke between non-vitamin K orally administered anticoagulants (NOACs) and aspirin. Awaiting the results of these trials and possible amending of the guidelines, the diagnosis of AF after cryptogenic stroke remains to have therapeutic consequences [3]. The diagnostic method, therefore, remains important.

In the CRYSTAL-AF trial, an implantable cardiac monitor (ICM) showed significantly higher detection rates of AF after 6-month follow-up in patients with cryptogenic stroke. However, placement of an ICM is costly and has some limitations, as it brings in the risk of pocket infection [6].

In the EMBRACE trial, a 30-day, event-triggered, loop recorder showed significantly higher rates of AF after 90 days of follow-up. Indeed, the device was moderately well-tolerated as 60% of all participants completed the full month of wearing the device [7].

In the past 5 years, smartphone-connected ECG devices have been developed. One of these devices is the Kardia Mobile (Alivecor, Inc., San Francisco, CA, USA). This a handheld ECG device that transmits and stores a single-lead ECG on a smartphone. It has been cleared by the United States Food And Drug Administration (FDA) and has received a European Union CE mark for the detection of AF [9]. The device is easy-to-use, non-invasive, electrically safe and can be used on demand. It does not introduce the risk of pocket infection, does not have to be worn on the body and is cheaper than an ICM [10, 11]. Furthermore, it does not, in contrast with an ICM, necessitate trained health care staff or a dedicated hospital room to hand the device to the patient [11, 12]. Therefore, the Kardia Mobile may be a more feasible alternative for prolonged ECG monitoring in cryptogenic stroke patients. However, the clinical effectiveness of the Kardia Mobile in detecting AF in cryptogenic stroke patients has not been investigated before.

Therefore, the Mobile phones in cryptogenic strOke patients Bringing sIngle Lead ECGs to detect Atrial Fibrillation (MOBILE-AF) trial is designed to investigate the effectiveness of the Kardia Mobile device for AF detection in patients with cryptogenic stroke or TIA and to compare this with the effectiveness of regular follow-up for AF detection.

In this paper the design and rationale of the study are presented.

## Methods

### Patient population

For this study, patients with cryptogenic stroke or TIA who have been treated at one of the participating centres (see the list in Additional file 1) will be asked to participate. Ischaemic stroke will be defined as an episode of neurological dysfunction caused by focal brain or retinal ischaemia with recent (hours to days) infarction on cerebral imaging [13]. A TIA is defined as a transient episode of neurological dysfunction lasting for less than 1 h caused by focal brain or retinal ischaemia without recent infarction on cerebral imaging [14].

A stroke or TIA is defined as cryptogenic if no cause can be determined after standard work-up, consisting of at least:

1. Computed tomography (CT) of the brain
2. Computed tomography angiography (CTA) of the head and neck arteries or echo Doppler of the carotid arteries
3. Transthoracic echocardiography followed by transoesophageal echocardiography when indicated
4. 12-lead 10-s electrocardiography
5. 24-h ECG monitoring
6. Laboratory tests:
  - Complete blood count
  - Prothrombin time
  - Partial thromboplastin time
  - Serum electrolytes
  - C-reactive protein
  - Hepatic and renal biochemical analysis
  - Erythrocyte sedimentation rate

If a stroke or TIA is considered to be cryptogenic, a patient will be evaluated with the described inclusion and exclusion criteria. These criteria are listed in Table 1. Generally, adult patients who suffer from a cryptogenic stroke or TIA, who are willing to sign informed consent, and are in possession of a smartphone with Android OS or iOS can participate. A maximum duration of 6 months between diagnosis of the index event and study inclusion is allowed.

**Table 1:** Inclusion and exclusion criteria

Inclusion criteria	
Admitted to the stroke unit of participating centres with an ischaemic stroke or TIA	
Exclusion criteria	
Known aetiology of TIA or ischaemic stroke	
Signs and symptoms mimicking TIA or ischaemic stroke that are caused by spinal ischaemia	
TIA only presenting with non-localising symptoms [25]	
Uncertainty about the diagnosis of TIA because of unclear clinical symptoms	
Myocardial infarction < 1 month before stroke	
Coronary artery bypass grafting < 1 month before stroke	
Surgery indicated valvular heart disease	
Documented history of AF or atrial flutter	
Left ventricular aneurysm on echocardiography	
Intracardiac thrombus on echocardiography	
Renal dysfunction (creatinine clearance < 30 mL/min/1.73 m^2^)	
Patient is not able or willing to sign the Informed Consent Form	
Patient is < 18 years of age	
Patient is considered an incapacitated adult	
Patient is not in possession of a smartphone or tablet with an Android Operating (OS) System or iOS and is unwilling to obtain one	

*AF* trial fibrillation, *TIA* transient ischaemic attack

### The Kardia Mobile

The Kardia Mobile device is a handheld smartphone-compatible device which contains two electrodes. The Kardia Mobile device is battery powered and electrically safe. The Kardia Mobile device communicates with the Kardia Mobile app, which can be downloaded on smartphones running Android OS or iOS. The device records a 30-s ECG from the fingers of both hands. A single-lead ECG is instantly shown on the smartphone screen. The recording of an ECG does not require the presence of health care staff.

An automated algorithm on the Kardia Mobile app checks the ECG for RR wave irregularity. It delivers a diagnosis citing either ‘no abnormalities detected,’ ‘possible atrial fibrillation’ or ‘this ECG could not be interpreted.’ This is a validated, FDA-approved algorithm with a 97% sensitivity and 98% specificity for AF detection [10].

After the diagnosis, the Kardia Mobile app offers the possibility to take notes. Patients are explicitly asked to take notes of their symptoms, if present, at the time of the recording.

**Fig. 1:**
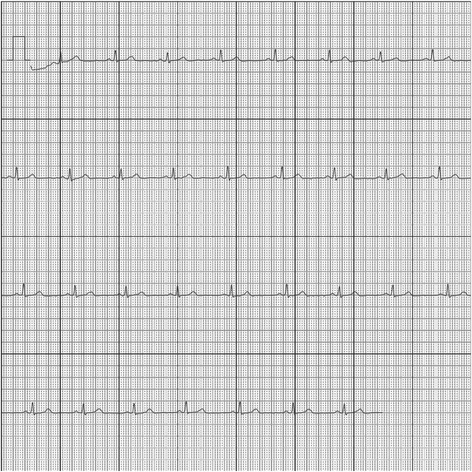
An electrocardiogram (ECG) recorded by the AliveCor device showing sinus rhythm

**Fig. 2:**
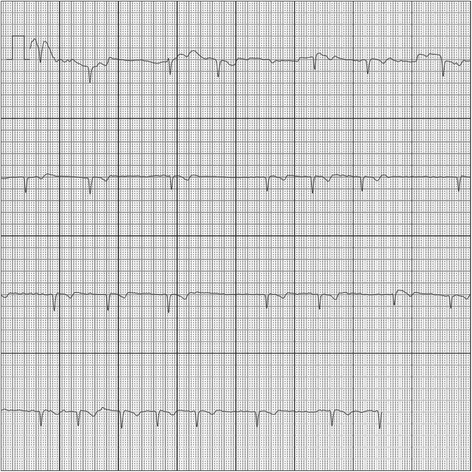
An electrocardiogram (ECG) recorded by the AliveCor device showing atrial fibrillation

If the diagnosis ‘possible atrial fibrillation’ or ‘this ECG could not be interpreted’ is delivered, the ECG is assessed for the presence of AF on the same or next working day by a PhD student with ample training and experience at the Holter department, who is not blinded to patient data. In case of any uncertainty about the diagnosis, the ECG is evaluated by an experienced cardiologist/electrophysiologist, who is blinded to the patient data. The ECG is saved in a secured cloud environment. ECGs in PDF can be automatically send to, and checked by, the study supervisors after patients’ consent. An example of such a PDF showing sinus rhythm is presented in Fig. 1. An example of such a PDF showing AF is presented in Fig. 2. The Kardia Mobile can be discontinued on a patient’s request. In case of non-adherence (defined as not having sent a single-lead ECG for two consecutive days), patients will first receive a standardised email asking if there are any technical problems. If patients do not send ECGs for another two consecutive days, they will receive a phone call asking for the reason. Technical issues will be addressed immediately. In case of loss of the Kardia device, a new device will be provided free of charge. If patients wish to discontinue their recordings, they will be considered lost to follow-up.

### 7-day Holter monitor

For this study, H3+ recorders (Mortara Instrument, Milwaukee, WI, USA) are deployed. All Holter recorders are battery powered and electrically safe. The Holter recorders have a CE mark. The H3+ recorder records three ECG channels continuously. A total of five electrodes are applied to the patient’s chest and abdomen. ECG data will be downloaded and analysed using the H-Scribe Holter Analysis System (Mortara Instrument, Milwaukee, WI, USA). This software package analyses the ECG for RR wave irregularities and automatically detects possible abnormalities such as premature atrial contractions, premature ventricular contractions, supraventricular arrhythmias and ventricular arrhythmias. All Holters are checked by a PhD student (who is not blinded to patient data) with ample training and experience at the Holter department, supervised by an experienced senior observer, who is blinded to the patient data. The 7-day Holter monitor can be discontinued on a patient’s request or in case of serious allergic reactions to the patches.

### Study design, randomisation and follow-up

**Fig. 3:**
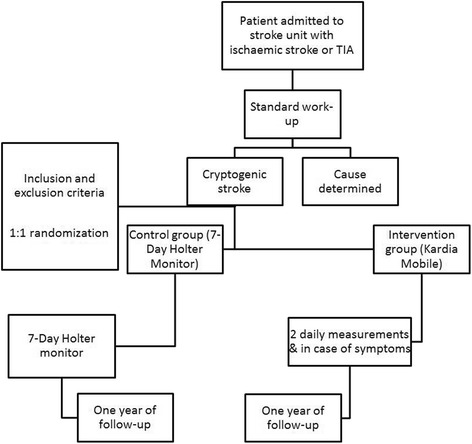
Flowchart of study design

**Fig. 4:**
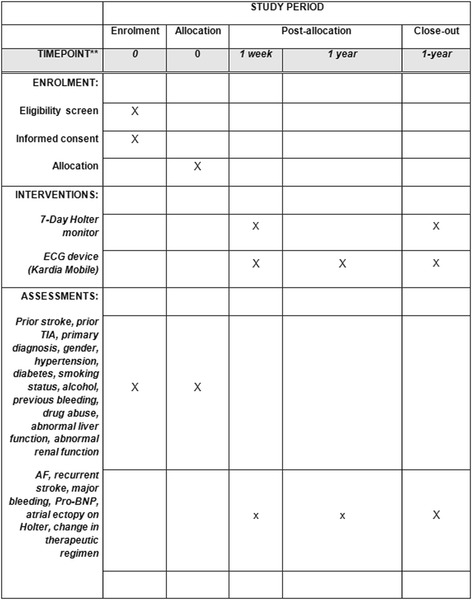
Standard Protocol Items: Recommendations for Interventional Trials (SPIRIT) Figure: schedule of enrolment, interventions and assessments

The Mobile phones in cryptogenic strOke patients Bringing sIngle Lead ECGs for Atrial Fibrillation detection trial (MOBILE-AF) is an international multicentre (a list of the six participating centres can be found in Additional file 1), randomised, open clinical trial, registered under clinical trial numbers NCT02507986 (https://www.clinicaltrials.gov/) and NL54103.058.15 (https://www.toetsingonline.nl/), in accordance with the Standard Protocol Items: Recommendations for Interventional Trials (SPIRIT) Checklist (in Additional file 2). A flowchart of the study design is given in Fig. 3 and the populated SPIRIT Figure on which this is based is shown in Fig. 4. It randomises patients, after inclusion, to follow-up with either the Kardia Mobile (intervention group) or the 7-day Holter monitor (control group). Block randomisation will be performed. Randomisation will be stratified per centre and per diagnosis (TIA or ischaemic stroke). The allocation sequence will be generated using a website (https://www.randomizer.org/) and stored in Excel (Microsoft, Redmond, WA, USA). The document is only accessible to a PhD student who is not otherwise involved in the trial. Patients will be approached and randomised by a project-dedicated PhD student. This PhD student will not have access to the allocation sequence.

When randomised to the intervention group, patients will receive the Kardia Mobile device. Patients will receive this device immediately after randomisation. They will be instructed by a dedicated PhD student about the usage of the Kardia Mobile, including downloading the app, setting up an account and recording an ECG with the Kardia Mobile device.

Patients are requested to record an ECG at least twice daily, with the two measurements separated by at least 6 h. Furthermore, patients are urged to record an ECG in case of any symptoms of possible cardiac origin, as judged by the patient.

When randomised to the control group, patients’ heart rhythm will be recorded for seven consecutive days (24 h per day), using the 7-day Holter monitor. This will be done directly after randomisation. After 7 days, patients are referred to regular follow-up after ischaemic stroke or TIA. Regular follow-up usually consists of a referral to the general practitioner who will see patients only in case of symptoms. However, patients might also be followed up at the site of referral, in which case a patient might have one more scheduled visit to the cardiologist. This is left to the discretion of the treating cardiologist.

In case AF is detected, the recording is sent to the treating cardiologist. The treatment options, including the decision to change anticoagulation treatment, will be left to the discretion of the treating cardiologist.

Patients will continue to participate in the trial, even though AF is documented. Each ECG that shows AF will be sent to the patient’s treating cardiologist.

All patients will continue to receive regular follow-up after cryptogenic stroke or TIA. All study actions are additional to regular follow-up. For both groups at 12 months, a phone call will be scheduled including a standardised interview (Additional file 3). If a patient has been admitted to a hospital other than the participating centre, the patient and hospital will be asked to share all relevant health information.

### Study outcomes

The primary outcome of this study is the percentage of patients in which AF is detected in the first year after the index ischaemic stroke or TIA. The primary outcome will be assessed by two cardiologists who will be blinded to patient data. Both will be working independent from each other. Any disagreements will be solved by consensus. AF is defined as a rhythm with completely irregular RR intervals and no distinct P waves on the surface ECG [15].

Secondary outcomes are

1. Pro-B-type natriuretic peptide (BNP) levels in all patients within 24 h after cryptogenic stroke
2. Percentages of atrial ectopy detected on the 24-h Holter monitor
3. Left atrial diameter and volume on 2D-echocardiography
4. Percentage of patients on orally administered anticoagulation therapy at the beginning and at the end of the study
5. The incidence of recurrent ischaemic stroke or TIA. Ischaemic stroke will be defined as an episode of neurological dysfunction caused by focal brain or retinal ischaemia with recent infarction on cerebral imaging [13]. A TIA is defined as a transient episode of neurologic dysfunction lasting less than 1 h caused by focal brain or retinal ischaemia without recent infarction on cerebral imaging [14]. All recurrent ischaemic strokes and TIAs will be evaluated by two neurologists based on information noted in the electronic health records, working independently of each other. Any disagreements will be solved by consensus
6. Major bleeding (defined as: any bleeding that needs medical treatment or hospital admission/prolongation of admission)
7. Number of single-lead ECGs taken per patient, as a measure of compliance
8. Number of Holter studies (either 24-h, 48-h or 7-day) done after randomisation in both groups (in which the 7-day Holter monitoring that is done as part of the trial protocol is not included)
9. Time (in days) between randomisation and detection of first AF episode

### Statistical analysis

The power calculation was done in PASS (Hintze J. (2008). PASS 2008. NCSS, LLC, Kaysville, UT, USA,. https://www.ncss.com/) and is based on a comparison of two proportions of patients with diagnosed AF in a two-by-two table with a chi-squared statistic or odds ratio calculated. The underlying assumption is that in 2% of the 7-day Holter monitor group AF will occur. This proportion is assumed to be 12.4% in the Kardia Mobile group. These percentages are based on the results of the CRYSTAL-AF trial [6]. A sample size of 200 patients was calculated with an alpha level of 0.05 and a power of 0.85. Patients will be 1:1 randomised.

Data will be analysed according to the intention-to-treat principle. After completion of the inclusion of study patients, the proportion of patients with AF will be compared with a chi-squared test or rate ratio (RR). Causes of missing data will be tabulated. Because of the expected low percentage of missing data, complete case analysis will be done. Analysis will be based on the missing-at-random assumption. In case of serious imbalances of baseline variables after randomisation, additional Poisson regression might follow with correction for potential confounding variables at baseline.

### Ethical conduct

The study will be conducted according to the principles of the Declaration of Helsinki (version 10, October 2013) and in accordance with the Dutch Medical Research Involving Human Subjects Act (WMO) and Good Clinical Practice guidelines. Potential study subjects will be approached by a project-dedicated PhD student who is not involved in their treatment. Written informed consent will be obtained from all study participants before randomisation (a translation of the informed consent form is given in Additional File 4). For this study, no data monitoring committee (DMC) is installed because both devices are battery powered and electrically safe; they do not bring risks to patients safety. The absence of a DMC is approved by the hospital’s Medical Ethics Committee (MEC). All devices used bear a CE mark and are approved by the United States FDA. No manufacturer of any devices used in this study is involved in study design, data collection, data analysis, data interpretation or writing of the report. No financial support or any other form of support is received for this study from any manufacturer. If the protocol changes such that it affects participants who are already participating in the trial, they will be notified by telephone or email by a project-dedicated PhD student. It is our intention to publish the results of the trial in a peer-reviewed scientific journal.

### Data safety

Data Collection Forms, Case Report Forms, Informed Consent Forms, and all other study documentation containing subject information, will be stored under locked conditions when not in use. Computers and all storage devices containing study data will be password-protected. Data stored on the computer will use an numeric code to identify the subject. Personal information will be kept in a password-protected, separate document. Access to data is restricted to study personnel and when required the MEC or the Healthcare Inspectorate as required by Dutch law. No personnel of any manufacturer of any device involved in the study will have access to the study data.

### Timeline

The sample size is 200 patients. Our trial started on 29 July 2016. Our proposed end date is 1 July 2019. Currently, six hospitals are referring patients for the trial. Each centre has an estimated 60 patients per year that are eligible for this study. In case of slow recruitment (fewer than 80 patients in 1 year), more hospitals will be approached to refer patients for participation in the trial. This request will be communicated via ClinicalTrials.gov. A final list of centres that referred patients to the trial will be published when the final results of the trial are available.

## Discussion

The MOBILE-AF trial is an international, multicentre, randomised clinical trial that evaluates the efficacy of the Kardia Mobile device in the detection of AF in cryptogenic stroke and TIA patients. To our knowledge, this is the first and only clinical trial that uses the Kardia Mobile for this indication. The Kardia Mobile is a validated device that is non-invasive and easy to use. Because of its negligible burden on the patient, its low cost and the fact that it can be used by patients on demand, without the presence of trained health care staff, in our opinion it has potential to improve the yield and cost-effectiveness of AF detection in this population.

### Subclinical AF after cryptogenic stroke and subsequent risk of recurrent stroke

Currently, there is scientific uncertainty about the causal relationship between subclinical paroxysmal AF following cryptogenic stroke and the subsequent risk of recurrent stroke [8, 16–19]. In contrast, clinical AF has been a long-known independent risk factor for ischaemic stroke [2]. More recent trials in patients without prior ischaemic stroke and implanted pacemakers or ICDs demonstrated that subclinical paroxysmal AF also increased ischaemic stroke risk [20, 21]. One of these trials, the ASSERT trial, found a 2.5-fold increased risk in patients who experienced episodes of subclinical AF lasting more than 6 min. This risk tended to increase in patients who experienced longer or more frequent episodes of subclinical paroxysmal AF. However, the authors noted that the study was underpowered to draw conclusions about this particular question [20].

### Detecting subclinical AF after cryptogenic stroke

In the CRYSTAL-AF trial, 12.4% of paroxysmal AF was found after 12 months in patients wearing an ICM. Of these episodes, 79% were asymptomatic. The median value of maximum duration of an AF episode was 11.2 h. A total of 61% of all AF episodes was longer than 6 h [6]. In the EMBRACE trial no data on AF symptoms are given. Of the 284 patients randomised to the 30-day ECG monitoring, 56 (19.7%) had AF of any duration. A total of 44 patients (15.5%) had at least one episode which lasted longer than 30 s. A total of 28 patients (9.9%) had at least one episode which lasted longer than 2.5 min [7].

The Kardia Mobile produces a PDF in which 30 s of measurement are shown. Therefore, the duration of an paroxysmal AF episode cannot be adequately determined by the Kardia Mobile. However, we consider it likely that a 30-s recording of AF on the Kardia Mobile will be part of a longer during episode of AF. In comparison, in the CRYSTAL-AF trial 61% of patients with detected AF had episodes which lasted longer than 6 h [6]. As we expect that most patients will perform a Kardia Mobile measurement twice daily, the chances that the Kardia Mobile will detect sporadically occurring AF episodes lasting only several minutes are low.

### Prevention of recurrent stroke

Determining the percentage of anticoagulants users, the percentage of recurrent strokes and the percentages of major bleedings are secondary objectives of the MOBILE-AF trial. At the end of the CRYSTAL-AF TRIAL, 10.1% in the ICM group and 4.6% of all control group patients used OAC. Recurrent stroke occurred in 5.2% in the ICM group and 8.6% in the control group. These results may indicate that OAC in patients with subclinical AF indeed lowers the risk of recurrent stroke. However, no data were shown about the relationship between duration or frequency of AF episodes and recurrent stroke. Furthermore, the study was underpowered to draw conclusions about this specific relation [6]. It therefore remains unclear whether subclinical AF in cryptogenic stroke is of clinical importance. For this reason, we leave the decision to start OAC to the discretion of the patient’s treating cardiologist. We will, however, carefully monitor percentages of patients in which OAC was described, as well as the main effect (stroke recurrence) and side effect (major bleedings) and, therefore, included these in our secondary objectives.

### Prediction of AF occurrence

There are a number of publications available about prediction of the occurrence of mainly paroxysmal AF after cryptogenic stroke or TIA. One is a paper by Rodriguez-Yanez et al., who evaluated 372 patients with cryptogenic stroke. Pro-BNP levels were determined within 24 h of stroke onset. Patients were followed-up for 2 years for the development of AF. The authors concluded that a pro-BNP level ≥ 360 pg/mL had a negative predictive value of 98.6%. Overall, 5.6% of all stroke patients were found to have AF [22]. This is a relatively low percentage, compared with the CRYSTAL-AF (12.4% after 1 year) and EMBRACE (16.2% after 90 days) [6, 7]. This might be explained by the frequency of monitoring: follow-up was done by taking elective 12-lead 10-s ECGs. No continuous monitoring was applied [22]. Therefore, as our trial involves more frequent ECG monitoring, we would like to corroborate that a pro-BNP level ≥ 360 pg/mL has indeed a high negative predictive value.

A second is a paper by Gladstone et al., who evaluated 237 patients with cryptogenic stroke or TIA. They assessed the number of atrial premature beats (ABP) on the 24-h Holter monitoring which was part of the standard work-up of cryptogenic stroke. They found that the number of ABPs was strongly and independently associated with the development of subclinical AF within 90 days after cryptogenic stroke [23]. We would like to also confirm these findings in our population.

A third is a paper by Tsang et al. [24], who investigated the relationship between left atrial diameter and volume and the development of AF. They found that an increase in left atrial volume was independently associated with the development of AF. Although this study was not performed in patients who had experienced a cryptogenic stroke, we believe that this might also be true for our population. We therefore would like to verify the study’s findings in our population [24].

To our knowledge, these three predictors have not been combined into a prediction score yet. We would like to combine the three predictors and develop a prediction score for the occurrence of paroxysmal AF after cryptogenic stroke or TIA in order to individualise monitoring strategies for these patients.

Summarising, we present a study that is designed to investigate the effectiveness of the Kardia Mobile to detect AF in patients with cryptogenic stroke. The Kardia Mobile is a device with serious potential to improve clinical and cost-effectiveness. The first results can be expected end 2018.

## Additional files

Additional file 1: List of participating centres. (DOCX 13 kb)

Additional file 2: SPIRIT 2013 Checklist: recommended items to address in a clinical trial protocol and related documents*. (DOC 121 kb)

Additional file 3: Standardized interview. (DOCX 15 kb)

Additional file 4: Atrial fibrillation after ischaemic stroke (translated from Dutch). (DOCX 16 kb)
