# Supplementary material for: Mobile phones in cryptogenic strOke patients Bringing sIngle Lead ECGs for Atrial Fibrillation detection (MOBILE-AF): study protocol for a randomised controlled trial
Source: Trials. 2017 Aug 29;18:402. doi: 10.1186/s13063-017-2131-0 (PMC5576132; doi:10.1186/s13063-017-2131-0)
Supplement: Supplementary file 1 — List of participating centres. (DOCX 13 kb) [file 13063_2017_2131_MOESM1_ESM.docx]

Additional file 1

**List of referring centers**

| **Name of hospital** | **Place** | **Country** |
| --- | --- | --- |
| Leiden University Medical Center | Leiden | The Netherlands |
| Haaglanden Medical Center | The Hague | The Netherlands |
| Groene Hart Hospital | Gouda | The Netherlands |
| Alrijne Hospital | Leiderdorp | The Netherlands |
| Regionshospitalet Herning | Herning | Denmark |
| Reinier de Graaf Hospital | Delft | The Netherlands |

**List of authors**

Roderick W. Treskes contributed to conception of design, drafted manuscript, gave final approval

Willem Gielen contributed to conception of design, critically revised the manuscript, gave final approval

Marieke JH Wermer contributed to conception of design, critically revised the manuscript, gave final approval

Robert W Grauss contributed to conception of design, critically revised the manuscript, gave final approval

Anouk P van Alem contributed to conception of design, critically revised the manuscript, gave final approval

Reza Alizadeh Dehnavi contributed to conception of design, critically revised the manuscript, gave final approval

Charles JHJ Kirchhof contributed to conception of design, critically revised the manuscript, gave final approval

Enno T van der Velde contributed to conception of design, critically revised the manuscript, gave final approval

Arie C Maan contributed to conception of design, critically revised the manuscript, gave final approval

Ron Wolterbeek contributed to conception of design, critically revised the manuscript, gave final approval

Onno M Overbeek contributed to conception of design, critically revised the manuscript, gave final approval

Martin J Schalij contributed to conception of design, critically revised the manuscript, gave final approval

Serge AIP Trines contributed to conception of design, critically revised the manuscript, gave final approval
