# Supplementary material for: Mobile phones in cryptogenic strOke patients Bringing sIngle Lead ECGs for Atrial Fibrillation detection (MOBILE-AF): study protocol for a randomised controlled trial
Source: Trials. 2017 Aug 29;18:402. doi: 10.1186/s13063-017-2131-0 (PMC5576132; doi:10.1186/s13063-017-2131-0)
Supplement: Supplementary file 3 — Standardized interview. (DOCX 15 kb) [file 13063_2017_2131_MOESM3_ESM.docx]

**Additional file 3**

Standardized interview taken at one year

| Question | Answer |
| --- | --- |
| Have you been admitted with an ischaemic stroke the last year? | - Yes - No |
| (if the previous question is answered with yes)  Would you allow us to ask the clinic you were admitted for the imaging (e.g. CT, CTA, MRI, etc) | - Yes - No |
| Have you been admitted with a TIA the last year? | - Yes - No |
| (if the previous question is answered with yes)  Would you allow us to ask the clinic you were admitted for the imaging (e.g. CT, CTA, MRI, etc) | - Yes - No |
| Have you been diagnosed with atrial fibrillation the last year? | - Yes - No |
| (if the previous question is answered with yes)  Would you allow us to ask the doctor that diagnosed you for the ECG? | - Yes - No |
| Could you send us your most recent medication list? | - Yes - No |
| (If the previous question is answered with no) Would you allow us to ask your pharmacy for a up-to-date medication list? | - Yes - No |
| Have you visited a doctor for a bleeding (e.g. nose bleeding or intracranial bleeding) last year? | - Yes - No |
| Have you had any holter monitoring (either 24, 48 or 7-day) after you were included in the mobile-af trial? | - Yes - No |
| (if the previous question is answered with yes)  How many have you had?  When have you had them? | …. times  DD/MM/YYYY |
