# Supplementary material for: Mobile phones in cryptogenic strOke patients Bringing sIngle Lead ECGs for Atrial Fibrillation detection (MOBILE-AF): study protocol for a randomised controlled trial
Source: Trials. 2017 Aug 29;18:402. doi: 10.1186/s13063-017-2131-0 (PMC5576132; doi:10.1186/s13063-017-2131-0)
Supplement: Supplementary file 4 — Atrial fibrillation after ischaemic stroke (translated from Dutch). (DOCX 16 kb) [file 13063_2017_2131_MOESM4_ESM.docx]

**Atrial fibrillation after ischaemic stroke (translated from Dutch)**

Dear sir/madam,

We kindly ask you to participate in medical-scientific research. Before you take a decision, please discuss this with family, friends or partners. We advise you to carefully read this instruction letter. If you have any questions, please feel free to ask the local investigator.

1. **What is the purpose of this research?**

Atrial fibrillation is a rhythm disorder of the heart in which the atria do not contract properly. Atrial fibrillation can cause a formation of a blood clot. If this blood clot blocks an artery that supplies the brain, an ischaemic stroke or TIA can occur. This risk can be decreased by prescribing oral anticoagulation. This can only be described if atrial fibrillation is detected. Undiagnosed atrial fibrillation can increase the risk of a recurrent stroke.

Recent research shows that atrial fibrillation can remain undiagnosed in patients with cryptogenic stroke or TIA. The purpose of this research is therefore to find out if a new device can increase the chance of finding atrial fibrillation. We want to compare this device with a 7-Day Holter.

1. **Which medical device is the topic of this research?**

The medical device is called the Kardia mobile, a mobile ECG device. This device allows you to make an ECG with your smartphone or tablet. It will take you approximately 30 seconds. Doctors can see from this ECG if you have atrial fibrillation or not. The use of the ECG device is safe.

1. **In what way is the research conducted?**

If you decide to participate, you will be randomized. You will receive either a Holter (a device that will record your rhythm for 7 consecutive days or a Kardia mobile. You will not receive both devices.

1. **What is expected when you participate?**

If you are randomized to the Holter, we kindly ask you to wear a 7-Day Holter monitor for 7 consecutive days. You will receive this device when you are in the hospital.

If you are randomized to the Kardia mobile, we kindly ask you to record two ECGs per day. We ask you to record an ECG if you experience symptoms of possible cardiac origin as well. You are welcome to record more ECGs per day if you want to.

1. **What is different than standard treatment?**

Both the 7-Day Holter monitor and the Kardia mobile are extra care. If you participate in this research, you will receive the same care as if you normally would. We will not deny you any tests or treatment because you participate.

1. **Which side effects can occur?**

Both the 7-Day Holter monitor and the Kardia mobile are safe to use. The 7-Day Holter monitor can be uncomfortable when you are sleeping.

1. **What are possible advantages and disadvantages of participating?**

An advantage is that you will receive additional monitoring to find out if you have atrial fibrillation. An early detection of atrial fibrillation with subsequent treatment might prevent a recurrent stroke or TIA. A disadvantage is that you have to go to the Leiden University Medical Center. Furthermore, you have to wear a Holter, or use a Kardia mobile, which might cause discomfort. Lastly, there is a small possibility that the Kardia incorrectly shows atrial fibrillation. This chance is however small: about 2%.

1. **What happens if you decide not to participate?**

You can decide if you participate or not. If you do not wish to participate, you have to do nothing. You do not have to sign anything. You also do not have to say why you do not want to participate. If you are a patient, your treatment will be continued. You can also decide to quit while participating, without saying why.

1. **What happens if the research is finished?**

Your regular care will be continued after you have participated.

1. **Are you insured when you participate?**

Every participant in this research is insured. The insurance covers damage that occurs as a consequence of the research. This accounts for any damage during and up to four years after participating in this research.

1. **Are you informed when relevant information will be available while the research is still going on?**

If you have a 7-day Holter monitor, you will receive a call if your Holter shows any relevant arrhythmias. If you have a Kardia mobile, it will give you a message after you recorded an ECG, which will say that you ECG is either “normal”, “possibly abnormal” or “undetermined”. We will check every ECG that is classified as “possibly abnormal” or “undetermined”. If an ECG is diagnosed as atrial fibrillation, we will notify your treating cardiologist.

1. **What will happen with your data?**

We are required by law to store your data for 15 years. If you participate in this research, you automatically give us permission to do so. If you do not want this, you cannot participate. All ECGs will be stored on the servers of Kardia mobile. Kardia mobile has to treat your data confidently. The company has a privacy statement and has to oblige by it.

1. **Is your general practitioner informed when you participate?**

We send a letter to your general practitioner when you participate. This is for your own safety. You have to give us permission when you participate. If you do not give permission, you cannot participate in this research.

1. **Will it cost you money to participate in this research?**

Both the Kardia mobile and the Holter are free of charge. Travel expenses will be reimbursed.

1. **Which medical ethics committee has approved this research?**

This research was approved by the Medical Ethics Committee of the Leiden University Medical Center.

1. **Do you have any questions?**

If you have any questions, please feel free to talk to the researcher or to the independent expert.
